# Neutralizing and protective murine monoclonal antibodies to the hemagglutinin of influenza H5 clades 2.3.2.1 and 2.3.4.4

**DOI:** 10.1111/irv.13152

**Published:** 2023-05-25

**Authors:** Carlotta Schuele, Falko Schmeisser, Megan Orr, Clement A. Meseda, Anupama Vasudevan, Wei Wang, Carol D. Weiss, Amy Woerner, Vajini N. Atukorale, Cyntia L. Pedro, Jerry P. Weir

**Affiliations:** ^1^ Laboratory of DNA Viruses, Division of Viral Products, Center for Biologics Evaluation and Research (CBER) Food and Drug Administration (FDA) Silver Spring Maryland USA

**Keywords:** A(H5N1), influenza, monoclonal antibody, protective epitopes

## Abstract

**Background:**

Highly pathogenic avian H5 influenza viruses have spread and diversified genetically and antigenically into multiple clades and subclades. Most isolates of currently circulating H5 viruses are in clade 2.3.2.1 or 2.3.4.4.

**Methods:**

Panels of murine monoclonal antibodies (mAbs) were generated to the influenza hemagglutinin (HA) of H5 viruses from the clade 2.3.2.1 H5N1 vaccine virus A/duck/Bangladesh/19097/2013 and the clade 2.3.4.4 H5N8 vaccine virus A/gyrfalcon/Washington/41088‐6/2014. Antibodies were selected and characterized for binding, neutralization, epitope recognition, cross‐reactivity with other H5 viruses, and the ability to provide protection in passive transfer experiments.

**Results:**

All mAbs bound homologous HA in an ELISA format; mAbs 5C2 and 6H6 were broadly binding for other H5 HAs. Potently neutralizing mAbs were identified in each panel, and all neutralizing mAbs provided protection in passive transfer experiments in mice challenged with a homologous clade influenza virus. Cross‐reacting mAb 5C2 neutralized a wide variety of clade 2.3.2.1 viruses, as well as H5 viruses from other clades, and also provided protection against heterologous H5 clade influenza virus challenge. Epitope analysis indicated that the majority of mAbs recognized epitopes in the globular head of the HA. The mAb 5C2 appeared to recognize an epitope below the globular head but above the stalk region of HA.

**Conclusions:**

The results suggested that these H5 mAbs would be useful for virus and vaccine characterization. The results confirmed the functional cross‐reactivity of mAb 5C2, which appears to bind a novel epitope, and suggest the therapeutic potential for H5 infections in humans with further development.

## 1 INTRODUCTION

Since the first detection in 1997, highly pathogenic avian H5N1 influenza viruses have continued to spread and diversify both genetically and antigenically^1^ with occasional human infection (https://cdn.who.int/media/docs/default‐source/influenza/human‐animal‐interface‐risk‐assessments/cumulative‐number‐of‐confirmed‐human‐cases‐for‐avian‐influenza‐a(h5n1)‐reported‐to‐who‐‐2003‐2023.pdf?sfvrsn=a11e93cf_1&download=true). Genetic analysis has resulted in the classification of H5 virus isolates into multiple clades and subclades,^2^ although most H5 viruses circulating in recent years have been in clades 2.3.2.1 or 2.3.4.4 (https://cdn.who.int/media/docs/default‐source/influenza/who‐influenza‐recommendations/vcm‐northern‐hemisphere‐recommendation‐2023‐2024/20230224_zoonotic_recommendations.pdf?sfvrsn=38c739fa_4). Depending on the public health risk assessment, candidate vaccine viruses (CVVs) derived from representative avian influenza viruses with pandemic potential are periodically developed for manufacturing inactivated vaccines as possible countermeasures (https://www.who.int/teams/global‐influenza‐programme/vaccines/who‐recommendations/zoonotic‐influenza‐viruses‐and‐candidate‐vaccine‐viruses). To facilitate characterization of emerging H5 influenza viruses and related CVVs, we have developed panels of mouse monoclonal antibodies (mAbs) to the influenza hemagglutinin (HA) of several H5 viruses. Here, we describe two panels of mouse mAbs generated to the HA of clade 2.3.2.1 and 2.3.4.4 H5 CVVs, H5N1 A/duck/Bangladesh/19097/2013 and H5N8 A/gyrfalcon/Washington/41088‐6/2014, respectively. These mAbs were characterized for hemagglutination inhibition (HI) and neutralizing activity in vitro and protection against virus challenge in vivo. Interestingly, one mAb in the 2.3.2.1 panel, designated as 5C2, did not have HI activity but was broadly neutralizing, not only for divergent clade 2.3.2.1 viruses but also H5 viruses from other H5 clades.

## 2 RESULTS

### 2.1 Isolation and characterization of mAbs to the HA of influenza H5 clades 2.3.2.1 and 2.3.4.4

We generated a panel of murine mAbs to the HA of a clade 2.3.2.1 H5N1 vaccine virus A/duck/Bangladesh/19097/2013 (Table 1, top). Six mAbs were selected that bound well to the homologous HA antigen used as the coating antigen in an ELISA. These six mAbs were further evaluated for neutralization of a lentiviral pseudovirus with the A/duck/Bangladesh HA and for virus HI using chicken red blood cells. Five of the six mAbs were neutralizing, but only four of the five neutralizing mAbs had HI activity. All six 2.3.2.1 mAbs worked well in Western blot analysis in nonreducing conditions; mAbs 1F3, 3D6, 6D9, and 6H6 also worked well in reducing conditions (Figure S1A).

**TABLE 1:** Characterization of H5 clades 2.3.2.1 and 2.3.4.4 mAbs.

A/dk/Bangladesh/19097/2013 mAbs	Isotype	ELISA binding^a^	Pseudovirus neutralization^b^	Hemagglutination inhibition^c^
1F3	IgG2b	0.00031	0.10	0.28
3D6	IgG3	0.00500	<0.08	4.42
4C12	IgG2a	0.00125	0.26	2.21
5C2	IgG2a	0.00125	0.39	>400
6D9	IgG2b	0.00016	0.08	0.10
6H6	IgG1	0.00250	>10	>400
A/gyrfalcon/Washington/41088‐6/2014 mAbs				
3H4	IgG2a	0.00063	<0.08	0.39
8D1	IgG2a	0.00063	0.06	0.55
5H3	IgG2b	0.00031	<0.08	>400
1C2	IgG2b	0.01563	0.90	1.11
1E1	IgG2b	0.00078	0.18	0.55

Abbreviation: mAbs, monoclonal antibodies.

^a^ The endpoint ELISA titer was defined as the lowest mAb concentration (μg/mL) that gave an absorbance value at 405 nm greater than 0.05.

^b^ Pseudovirus neutralization titer was defined as the lowest mAb concentration (μg/mL) that resulted in a 95% reduction of luciferase units of a retrovirus pseudotype expressing the homologous influenza hemagglutinin.

^c^ Hemagglutination inhibition titer was defined as the lowest mAb concentration (μg/mL) that inhibited the homologous influenza virus agglutination of chicken red blood cells.

A second panel of murine mAbs was generated to the HA of a clade 2.3.4.4 H5N8 vaccine virus A/gyrfalcon/Washington/41088‐6/2014 HA (Table 1, bottom). Five mAbs were selected that bound well to the homologous HA antigen used as the coating antigen in an ELISA. These five mAbs were further evaluated for neutralization of a lentiviral pseudovirus with the A/gyrfalcon/Washington HA and for inhibition of virus hemagglutination of chicken red blood cells. All five mAbs were neutralizing, and four of the five mAbs had HI activity. All five 2.3.4.4 mAbs detected HA in Western blot analysis but only in nonreducing conditions (Figure S1B).

### 2.2 Isolation of escape viruses and epitope identification for H5‐specific mAbs

To better understand the HA epitopes identified by the H5 neutralizing mAbs, virus escape mutants were selected by incubation of the virus with each of the neutralizing mAbs over a range of concentrations, followed by infection of Madin‐Darby Canine Kidney (MDCK) cells with the virus–antibody mixtures. Using virus obtained at the highest concentration of mAb, the process was repeated at a fourfold higher mAb concentration. Following approximately four rounds of neutralization/selection, the HA gene of potential escape viruses was sequenced, and the results compared with the sequence of the parent virus.

**FIGURE 1:**
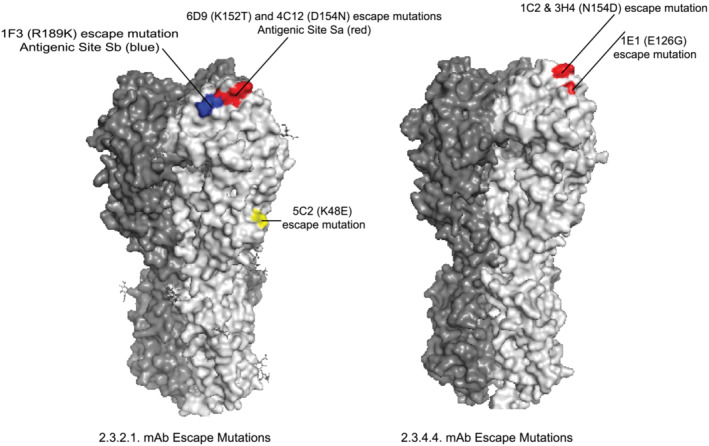
Location of hemagglutinin (HA) amino acid changes in influenza H5 escape mutants. (left) Location of escape mutants to H5 2.3.2.1 monoclonal antibodies on the HA trimer of a 2.3.2.1 virus HA (A/Hubei/1/2010 PDB ID: 4KTH). (right) Location of escape mutants to H5 2.3.4.4 monoclonal antibodies on the HA trimer of a 2.3.4.4 virus HA (A/gyrfalcon/Washington/41088‐6/2014 PDB ID: 5HUF).

A/duck/Bangladesh/19097/2013 vaccine virus escape mutants were isolated for mAbs 1F3, 4C12, 6D9, and 5C2. The 1F3 escape mutation R189K is located in antigenic site Sb (amino acid numbering refers to the mature HA, excluding the HA N‐terminal signal peptide) but is sterically close to the 4C12 and 6D9 escape mutations, D154N and K152T, respectively, that are in antigenic site Sa (Figure 1, left). Sequencing of an escape virus to mAb 5C2, which was neutralizing but did not have HI activity, identified a K48E mutation and a K377T amino acid change (position 381 in the wild‐type virus that does not have the polybasic region removed). Neither amino acid is located in one of the major known antigenic sites, and only amino acid 48 is located on the outer surface of the HA. A second independently obtained escape virus to 5C2 identified an S106R mutation, and although amino acid 106 is located on the inside of the HA, hidden from the exterior, it is spatially close to amino acid 48. These results suggest the epitope recognized by mAb 5C2 is below the globular head of the HA trimer but above the stalk region (Figures 1, left, and S2).

**FIGURE 2:**
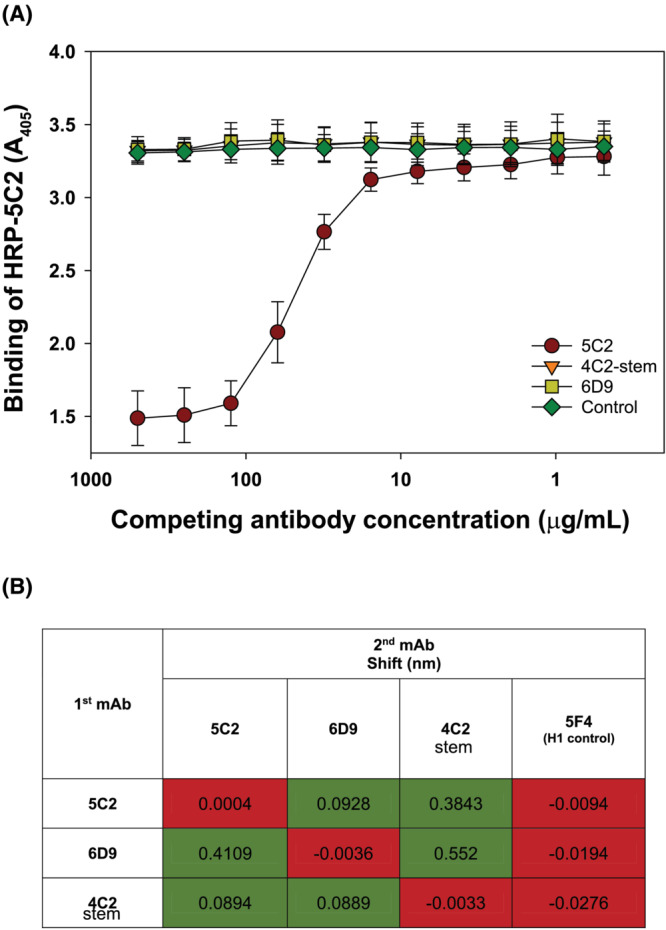
Binding competition between monoclonal antibody (mAb) 5C2 and hemagglutinin (HA) head and stalk‐binding mAbs. (A) ELISA competition. Plates were coated with recombinant A/duck/Bangladesh/19097/2013 HA and then incubated with dilutions of mAbs 5C2, 6D9, and 4C2‐stem and an unrelated control influenza mAb, at a starting concentration of 500 μg/mL, followed by incubation with horseradish peroxidase (HRP)‐labeled 5C2. (B) Biolayer interferometry competition. Ni‐NTA biosensors were coated with His‐tagged recombinant A/duck/Bangladesh/19097/2013 HA at 3 μg/mL. The mAbs 6D9, 5C2, and 4C2‐stem were loaded at a concentration of 30 μg/mL for initial saturating binding to HA and used at a concentration of 30 μg/mL as the competing mAb. The matrix table shows the shifts in wavelength following the second antibody association step. Green color (no competition) corresponds to cell values (wavelength shift) more than threefold over second step binding of homologous antibody.

A set of competition binding experiments was performed for further characterization of the binding of mAb 5C2. In an ELISA experiment, A/duck/Bangladesh/19097/2013 HA was incubated first with mAbs 5C2, 6D9 (head‐binding mAb), 4C2 (a stem‐binding mAb), and an unrelated control influenza mAb, followed by incubation with horseradish peroxidase (HRP)‐labeled 5C2 (Figure 2). Binding of labeled 5C2 was inhibited only by unlabeled 5C2 and not by the unrelated control mAb nor by the head or stem mAbs (6D9 and 4C2, respectively). These results were confirmed in biolayer interferometry experiments. When used as a first binding antibody, each of the three mAbs, 5C2, 6D9, and the stem‐binding 4C2, bound to A/duck/Bangladesh/19097/2013 HA immobilized on Ni‐NTA biosensors. When subsequently used as second binding antibodies, there was further binding of each mAb when any of the other mAbs had been used as the first binding mAb, indicating a lack of competition. Taken together, the results of the competition experiments indicate that mAb 5C2 binds to a different part of HA than the globular head (e.g., 6D9) or stem region (e.g., 4C2).

Two independent attempts were made to isolate A/gyrfalcon/Washington/41088‐6/2014 vaccine virus escape mutants, but all recovered escape viruses had the same A156T mutation that adds a glycosylation motif to the HA. Because addition of a glycosylation site has the potential to shield a large area of HA from interaction with mAbs, it may not offer useful information about the epitopes recognized by individual mAbs. As an alternative approach, we attempted to isolate escape mutants to the clade 2.3.4.4 mAb panel using a related 2.3.4.4 vaccine virus A/Sichuan/26221/2014. Using this approach, we isolated an N154D mutation to mAbs 1C2 and 3H4 and an E126G mutation to mAb 1E1. Both amino acids are in antigenic site Sa (Figure 1, right). An A156T mutation was again isolated to mAbs 5H3 and 8D1. Taken together, the results suggest that all five of the 2.3.4.4 mAbs recognize a region in the globular head of the HA near the antigenic site Sa.

### 2.3 mAb cross‐neutralization of escape viruses

The escape viruses to H5 2.3.2.1 and 2.3.4.4 mAbs were further characterized by plaque‐reduction neutralization titer (PRNT) assays against their respective mAb panel. All five of the 2.3.2.1 mAbs that were previously shown to have pseudovirus neutralizing activity were able to neutralize A/duck/Bangladesh/19097/2013 in a PRNT assay at concentrations from <0.3 to 5 μg/mL, but the mAbs did not neutralize their own escape virus, confirming the escape virus phenotype (Table 2, top). The mAb 5C2 neutralized the other three escape viruses, and the 5C2v escape virus was neutralized by each of the other four mAbs, indicating that the epitope recognized by 5C2 was different from epitopes recognized by the other mAbs in this panel. In contrast, the 1F3v, 4C12v, and 6D9v escape viruses were resistant to neutralization by all of the mAbs in this panel except for 5C2, indicating that epitopes recognized by 1F3, 3D6, 4C12, and 6D9 are close to each other on the HA and distant from the 5C2 epitope.

**TABLE 2:** mAb neutralization of H5 escape viruses.

Virus^a^	mAb concentration for 50% neutralization (μg/mL)^b^				
	1F3	3D6	4C12	5C2	6D9
A/dk/Bangladesh	<0.3	<0.3	1.25	5	<0.3
1F3v (R189K)	20	0.16	>320	0.6	80
4C12v (D154N)	>80	80	>80	1.25	>80
5C2v (K48E)	1.25	<0.3	5	>80	1.25
6D9v (K152T)	>80	5	>80	1.25	>80
	**1C2**	**1E1**	**3H4**	**5H3**	**8D1**
A/Sichuan	20	20	5	<0.3	1.25
1C2v (N154D)	>80	>80	>80	>80	>80
1E1v (E126G)	>80	>80	>80	>80	>80
3H4v (N154D)	>80	>80	>80	>80	>80
5H3v (A156T)	>80	>80	>80	>80	>80

Abbreviation: mAb, monoclonal antibody.

^a^ Each virus was titrated and diluted to approximately 500 pfu/mL and incubated with mAb concentrations from 80 to 0.3 μg/mL for incubation with mAb in a plaque‐reduction neutralization experiment.

^b^ All mAbs completely neutralized their parent virus at concentrations between 0.3 and 20 μg/mL (gray shading in the table); escape mutants that were neutralized by each mAb are also shown in gray.

The five 2.3.4.4 mAbs previously shown to neutralize A/gyrfalcon/Washington/41088‐6/2014 also were able to neutralize A/Sichuan/26221/2014 in a PRNT assay at concentrations from <0.3 to 20 μg/mL (Table 2, bottom). None of the mAbs neutralized the four available escape viruses, both confirming the escape virus phenotypes and indicating that the epitopes recognized by all five 2.3.4.4 mAbs are close to each other on the globular head of the HA. Interestingly, this includes mAb 5H3, which does not have HI activity.

### 2.4 Cross‐reactivity of mAb binding and neutralization

We investigated whether the two H5 mAb panels cross‐reacted with the HAs of other H5 clades using ELISA binding and pseudovirus neutralization assays (Table 3). From the A/duck/Bangladesh 2.3.2.1 mAb panel, mAbs 1F3, 3D6, 4C12, and 6D9 did not bind to HA antigens from clade 1, clade 2.1.3.2, clade 2.3.4, or clade 2.3.4.4, whereas mAbs 6H6 and 5C2 bound well to all of these HA antigens in an ELISA. None of the mAbs in the A/gyrfalcon/Washington 2.3.4.4 mAb panel bound to HA antigens from clade 1, clade 2.1.3.2, clade 2.3.4, or clade 2.3.2.1.

**TABLE 3:** Influenza H5 ELISA cross‐reactivity.

	H5 virus antigen^a^							
	A/Vietnam clade 1		A/Indonesia clade 2.1.3.2		A/Anhui clade 2.3.4		A/gyrfalcon clade 2.3.4.4c	
mAbs	ELISA^b^	PVN^c^	ELISA	PVN	ELISA	PVN	ELISA	PVN
1F3	>8	>10	>8	>10	>8	>10	>8	N.D.
3D6	2	>10	4	>10	1	>10	2	N.D.
4C12	>8	>10	>8	>10	>8	>10	>8	N.D.
5C2	0.0006	0.390	0.0006	0.060	0.0006	0.060	0.0050	N.D.
6D9	1	>10	4	>10	2	>10	>8	N.D.
6H6	0.0006	>10	0.0050	>10	0.0078	>10	0.0006	N.D.

	A/Vietnam clade 1		A/Indonesia clade 2.1.3.2		A/Anhui clade 2.3.4		A/duck/Bangladesh clade 2.3.2.1a	
3H4	>8	>10	>8	>10	>8	>10	>8	N.D.
8D1	>8	>10	>8	>10	>8	>10	>8	N.D.
5H3	>8	>10	>8	>10	>8	>10	>8	N.D.
1C2	>8	>10	>8	>10	>8	>10	>8	N.D.
1E1	>8	>10	>8	>10	>8	>10	>8	N.D.

Abbreviations: mAbs, monoclonal antibodies; N.D., not done; PVN, pseudovirus neutralization.

^a^ Virus antigens: A/Vietnam/1203/2004 (A/Vietnam); Indonesia/5/2005 (A/Indonesia); A/Anhui/1/2005 (A/Anhui); A/gyrfalcon/Washington/41088‐6/2014 (A/gyrfalcon); and A/duck/Bangladesh/19097/2013 (A/duck/Bangladesh).

^b^ The endpoint ELISA titer was defined as the mAb concentration (μg/mL) that gave an absorbance value at 405 nm greater than 0.05.

^c^ PVN titer was defined as the mAb concentration (μg/mL) resulting in a 95% reduction of luciferase units of a retrovirus pseudotype expressing the indicated influenza hemagglutinin.

When these mAbs were analyzed for neutralization of pseudoviruses with the HAs from clade 1, clade 2.1.3.2, and clade 2.3.4.4 H5 viruses, only mAb 5C2 was able to neutralize each of these pseudoviruses, whereas the other mAbs were not. Although this analysis was limited to a relatively small number of H5 HAs, the data suggest that most of the mAbs in each panel are clade‐specific. Only the clade 2.3.2.1 mAbs 6H6 and 5C2 appear to have good cross‐clade binding activity, and only 5C2 has cross‐clade neutralizing activity.

Because both clades 2.3.2.1 and 2.3.4.4 viruses have continued to evolve since the A/duck/Bangladesh/19097/2013 and A/gyrfalcon/Washington/41088‐6/2014 vaccine viruses were prepared, we tested each panel of mAbs for neutralization of other viruses in their respective clades. Because of the observed cross‐reactivity of mAb 5C2, we included this 2.3.2.1 mAb in the analysis of clade 2.3.4.4 vaccine viruses.

**FIGURE 3:**
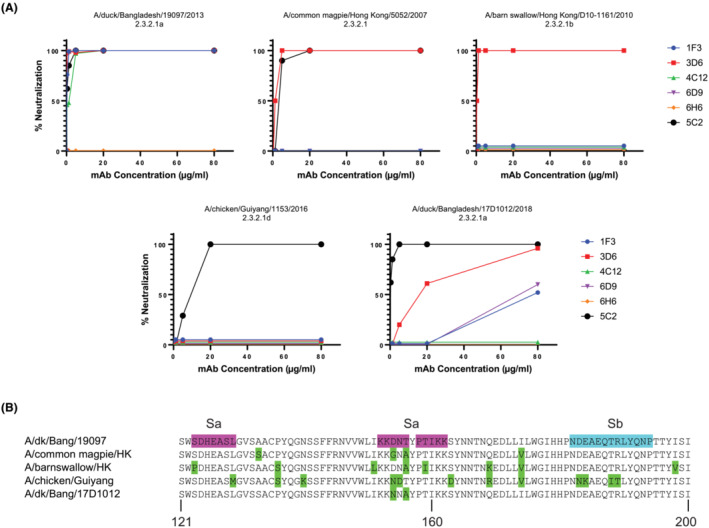
Plaque‐reduction neutralization of influenza H5 viruses with H5 2.3.2.1 monoclonal antibodies (mAbs). (A) Five 2.3.2.1 H5 viruses were incubated with dilutions of the indicated H5 mAbs at a starting concentration of 80 μg/mL prior to plaque assay with the indicated virus. (B) Amino acid sequences of the five 2.3.2.1 hemagglutinins in the antigenic region encompassing antigenic sites Sa and Sb. Amino acid differences compared with A/duck/Bangladesh/19097/2013 are shown in green.

The six 2.3.2.1 mAbs were tested for plaque neutralization of four additional H5 vaccine viruses from H5 2.3.2.1 subclades (Figure 3A). The HA amino acid sequences of these vaccine viruses in the Sa and Sb antigenic regions are shown in Figure 3B. Only mAbs 3D6 and 5C2 were able to neutralize A/common magpie/Hong Kong/5052/2007, only 3D6 was able to neutralize A/barn swallow/Hong Kong/D10‐11613212/2010, and only 5C2 was able to neutralize A/chicken/Guiyang/1153/2016. Four mAbs (1F3, 3D6, 6D9, and 5C2) were able to neutralize A/duck/Bangladesh/17D1012/2018, although 6D9 and 1F3 only at the highest concentration tested. The results indicate that all of the mAbs in the 2.3.2.1 panel have unique binding characteristics even though the escape virus characterizations (Figure 1 and Table 2) indicated that the four globular head‐binding mAbs recognized epitopes close to each other.

**FIGURE 4:**
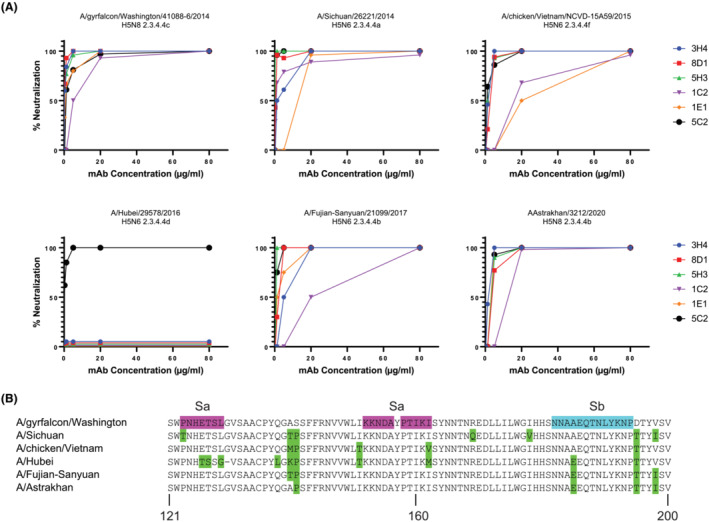
Plaque reduction neutralization of influenza H5 viruses with H5 2.3.4.4 monoclonal antibodies (mAbs). (A) Six 2.3.4.4 H5 viruses were incubated with dilutions of the indicated H5 mAbs at a starting concentration of 80 μg/mL prior to plaque assay with the indicated virus. (B) Amino acid sequences of the six 2.3.4.4 hemagglutinins in the antigenic region encompassing antigenic sites Sa and Sb. Amino acid differences compared with A/gyrfalcon/Washington/41088‐6/2014 are shown in green.

The five 2.3.4.4 mAbs, along with the cross‐reactive 5C2, were tested for plaque neutralization of five additional H5 vaccine viruses from H5 2.3.4.4 subclades (Figure 4A). The HA amino acid sequences of these vaccine viruses in the Sa and Sb antigenic regions are shown in Figure 4B. Clade 2.3.2.1 mAb 5C2 was able to neutralize all 2.3.4.4 vaccine viruses, confirming its cross‐reactive phenotype. The five 2.3.4.4 mAbs were able to neutralize the other 2.3.4.4 vaccine viruses to varying degrees, except for the subclade d virus A/Hubei/29578/2016.

### 2.5 Protection afforded by passive transfer of H5 HA mAbs

To assess the protective capacity of the H5 HA mAbs, mAbs or phosphate buffered saline (PBS) were passively transferred to BALB/c mice by intraperitoneal injection approximately 6 h before intranasal challenge with 10 LD_50_s of either an H5 2.3.2.1 or H5 2.3.4.4 virus. Successful setup of such challenge experiments requires that the H5 vaccine viruses used for challenge can be grown to a high titer and be sufficiently virulent in mice. Preliminary experiments determined that the A/duck/Bangladesh/19097/2013 and A/Sichuan/26221/2014 vaccine viruses were lethal in mice and established the LD_50_ for the challenge experiments.

**FIGURE 5:**
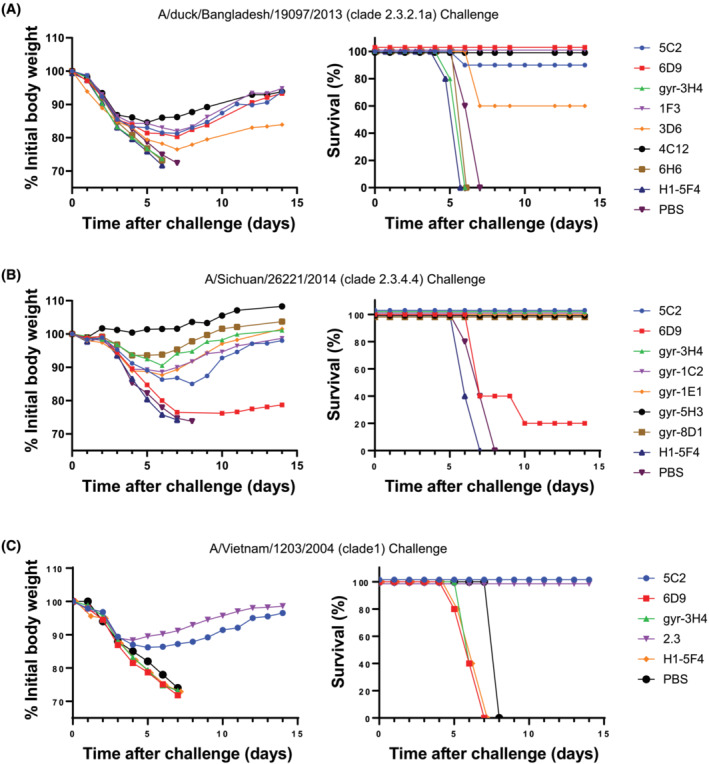
Protection of mice from influenza H5 challenge. PBS or the indicated monoclonal antibody (100 μg) were transferred by the intraperitoneal route into mice (groups of five) approximately 6 h before being challenged intranasally with the indicated H5 virus. Animals were observed for 2 weeks for average percentage change in body weight (left) and death (right). (A) A/duck/Bangladesh/19097/2013 challenge. (B) A/gyrfalcon/Washington/41088‐6/2014 challenge. (C) A/Vietnam/1203/2004 challenge.

All of the clade 2.3.2.1 mAbs except for 6H6, which did not have either neutralizing or HI activity, provided protection against homologous H5 2.3.2.1 virus challenge (Figure 5A). No protection was observed in the PBS group, a group of mice given an H1 control mAb (5F4) or a group of mice receiving an H5 mAb from the 2.3.4.4 mAb panel (3H4). Similarly, all of the clade 2.3.4.4 mAbs provided protection against homologous 2.3.4.4 virus challenge, as did the cross‐reactive mAb 5C2 from the 2.3.2.1 mAb panel (Figure 5B). Mice receiving PBS, an H1 control mAb (5F4), or another H5 mAb from the 2.3.2.1 mAb panel (6D9) were not protected.

An additional H5 challenge model, a clade 1 A/Vietnam/1203/2004 virus, was used to assess protection of the cross‐reactive mAb 5C2 (Figure 5C). The cross‐reactive mAb 5C2 provided protection against challenge with this heterologous virus, as did a clade 1 neutralizing mAb 2.3, but neither the 2.3.2.1‐specific mAb 6D9, the clade 2.3.4.4‐specific mAb 3H4, the H1 5F4 control mAb, nor PBS provided protection. The results confirm the functional cross‐reactivity of the novel epitope binding mAb 5C2.

## 3 DISCUSSION

In the study reported here, we developed and characterized panels of murine mAbs to the HA from the two most commonly circulating clades of influenza H5, clades 2.3.2.1 and 2.3.4.4. The primary goal of these studies was to provide reagents that would facilitate characterization of influenza viruses with pandemic potential and the related candidate vaccines developed as countermeasures to such viruses. The results from several types of analyses suggested that these H5 mAbs would be useful for assorted purposes of virus and vaccine characterization.

All of the selected mAbs, six 2.3.2.1 and five 2.3.4.4 mAbs, bound their homologous HA antigen well in an ELISA format. Only two mAbs from the 2.3.2.1 panel, 5C2 and 6H6, were not clade‐specific in this format; both mAbs bound HA from all other H5 clades tested, suggesting their usefulness as pan‐H5 ELISA reagents. Five of the six 2.3.2.1 mAbs and all five of the 2.3.4.4 mAbs potently neutralized their homologous virus, A/duck/Bangladesh/19097/2013 and A/gyrfalcon/Washington/41088‐6/2014, respectively, as shown in both live virus neutralization and pseudovirus neutralization assays. Interestingly, one neutralizing mAb from each panel, 5C2 and 5H3, did not have HI activity. Neutralization was correlated with protection in passive transfer experiments in mice; all mAbs with neutralizing activity were protective when mice were challenged with a homologous clade influenza virus.

Several methods were used to identify the HA epitopes recognized by the mAbs in the two panels. Isolation and sequencing of escape viruses, combined with mAb cross‐neutralization of escape viruses, indicated that the majority of the mAbs recognized epitopes in the globular head of the HA. Most neutralizing influenza antibodies that bind to the globular head of HA, and that also have HI activity, are usually strain‐specific. Five distinct antigenic sites on the HA have been defined and characterized, designated as either antigenic sites A–E using influenza H3^3^, ^4^ or Sa, Sb, Ca1, Ca2, and Cb using influenza H1,^5^, ^6^ a Group 1 HA as is H5.

Three escape mutants were isolated in the H5 equivalent antigenic sites Sa and Sb of the 2.3.2.1 virus A/duck/Bangladesh/19097/2013 using the mAbs from the 2.3.2.1 mAb panel. These mutations, K152T, D154N, and R189K, are physically close to each other on the head of the HA. Indeed, cross‐neutralization experiments with the escape mutant viruses and the 2.3.2.1 mAbs indicated substantial resistance of all escape viruses to neutralization by all neutralizing mAbs except 5C2, suggesting that mAbs 1F3, 3D6, 4C12, and 6D9 bind to a similar region in the HA near antigenic sites Sa and Sb. However, there are differences in the binding and neutralization mediated by these four 2.3.2.1 mAbs as shown by the neutralization results with viruses from other 2.3.2.1 subclades. For example, mAb 4C12 did not neutralize any of the other four tested 2.3.2.1 subclade viruses; mAbs 6D9 and 1F3 only neutralized the 2.3.2.1a virus A/duck/Bangladesh/17D1012/2018; and mAb 3D6 neutralized three of the four tested viruses, but not the subclade 2.3.2.1d virus A/chicken/Guiyang/1153/2016.

Three escape mutants, E126G, N154D, and A156T, were isolated in the H5 equivalent antigenic site Sa of the 2.3.4.4 virus A A/Sichuan/26221/2014 using the mAbs from the 2.3.4.4 mAb panel. Cross‐neutralization experiments with the escape viruses, as well as neutralization results with viruses from other 2.3.4.4 subclades, indicated that all of these mAbs in this panel bound to a similar region in the globular head of HA head. Interestingly, this included mAb 5H3, which did not have HI activity. The 2.3.4.4 mAbs neutralized several viruses representing other 2.3.4.4 subclades with the exception of the 2.3.4.4d virus A/Hubei/29578/2016. Of note, all of the 2.3.4.4 mAbs, as well as the cross‐reactive mAb 5C2, potently neutralized A/Astrakhan/3212/2020, a 2.3.4.4b virus from a recent human infection.^7^ In recent clinical trials evaluating a monovalent inactivated A/gyrfalcon/Washington/41088‐6/2014 vaccine adjuvanted with either AS03 or MF59, substantial cross‐reactive HI antibody responses to A/Astrakhan/3212/2020 and other 2.3.4.4 viruses were reported, but poor cross‐reactive HI responses to more distant H5 clade viruses were observed.^8^, ^9^

Additional characterization of mAb 5C2 was undertaken because of the interesting phenotype presented. This mAb, isolated as part of the panel of mAbs against 2.3.2.1 virus A/duck/Bangladesh/19097/2013, did not have HI activity but had broad HA binding and neutralizing activity against divergent clade 2.3.2.1 viruses, as well for H5 viruses from other H5 clades. In our studies, mAb 5C2 neutralized every H5 virus tested except for A/barn swallow/Hong Kong/D10‐1161/2010, but it appears to be H5‐specific because binding and neutralization was not observed with H1 viruses (data not shown). Comparison of the A/barn swallow/Hong Kong/D10‐1161/2010 HA sequence to that of A/duck/Bangladesh/19097/2013 (Figure S3) did not suggest an obvious explanation for the inability of 5C2 to neutralize the A/barn swallow virus. Epitope mapping by escape mutant isolation did not present a clear picture of the epitope recognized by 5C2. An S106R mutation from one escape mutant is not located on the outside of HA. A second escape mutant had two amino acid changes at K48E and K377T, the latter mutation also not located on the outside of HA. Nevertheless, the location of the K48E mutation, together with the results from competition experiments with head‐ and stem‐binding mAbs, suggests that mAb 5C2 recognizes an epitope below the globular head but above the stalk region of HA. Previously described influenza stem‐binding antibodies typically recognize many influenza subtypes, whereas the binding and neutralization properties of 5C2 appear broad, but only for other H5 viruses. Additional experimental approaches will be needed to more accurately define how 5C2 binds H5 influenza HA.

In summary, the murine mAbs described here should be useful reagents for characterization of influenza viruses with pandemic potential, as well as reagents for evaluating CVVs and candidate inactivated pandemic vaccines. For example, several previous studies have described the development of new assays that use mAbs for determining the potency of inactivated influenza vaccines.^10^ The mAbs described here should be useful for setup of H5‐specific potency assays. In particular, the cross‐reactive mAb 5C2 may be an especially versatile antibody reagent. This mAb, or antibodies identified with similar specificities, may also have therapeutic potential for H5 infections in humans if developed as a humanized antibody.

## 4 MATERIALS AND METHODS

### 4.1 Cells and viruses

The H5 viruses used in these studies were reassortant CVVs (http://www.who.int/influenza/vaccines/virus/en/). Influenza viruses were propagated in either 9‐day‐old specific pathogen‐free embryonated chicken eggs or MDCK cells. Virus titer was determined by plaque assay on MDCK cells. All experiments, including animal experiments, were conducted at biosafety level 2 plus laboratory conditions.

### 4.2 Monoclonal antibodies

mAbs to H5 HA were prepared as previously described.^11^ BALB/c mice were immunized and boosted with mammalian‐derived influenza virus‐like particles containing the influenza HA from either A/duck/Bangladesh/19097/2013 or A/gyrfalcon/Washington/41088‐6/2014. All mAbs were purified and concentrated by protein G chromatography for use in the experiments described. The stem‐binding mAb 4C2 and control H1 mAbs have been described previously.^11^, ^12^

### 4.3 Animals and immunizations

All immunizations and blood draws were performed in accordance with an animal study protocol approved by the FDA White Oak Consolidated Animal Program. Six‐ to eight‐week‐old male BALB/cByJ mice were purchased from the Jackson Laboratories (Bar Harbor, Maine) and housed in cages at a core facility at CBER/FDA. Sterile food and water were supplied ad libitum. All antibody transfers and challenges were performed in accordance with an animal study protocol approved by the CBER/FDA Animal Care and Use Committee (#2008‐02); procedures were similar to those described previously.^13^ mAbs (100 μg/mouse in 0.5‐mL PBS) were delivered by intraperitoneal injection; for virus challenge, each mouse received 10 μL of virus suspension in the naris of each nostril while under anesthesia (intraperitoneal injection of 0.1 mL/20‐g body weight of a mouse cocktail of ketamine/xylazine [ketamine = 100/mg/mL, xylazine = 20 mg/mL]). Mice were weighed daily thereafter and monitored for 2 weeks. Any mouse that lost 25% of body weight (the study endpoint) at any time point was sacrificed according to the approved animal protocol and was considered to have succumbed to infection.

### 4.4 Preparation of soluble A/duck/Bangladesh/19097/2013 HA

Soluble HA was prepared by baculovirus expression in insect cells essentially as described by others.^14^ The synthetic DNA sequence encoding the ectodomain of the HA of H5N1 strain A/duck/Bangladesh/19097/2013 was cloned downstream of the sequence encoding the GP67 signal peptide and upstream of the sequences for the T4 phage foldon trimerization signal and 6x His‐tag to generate a recombinant bacmid for protein production in a Baculovirus expression system. The bacmid was transfected into Sf9 insect cells to generate an infectious recombinant Baculovirus stock, and the recombinant virus was then used to infect High Five insect cells for protein expression. Supernatant was harvested 72–96 h post infection, and recombinant His‐tag containing protein was purified using Ni‐NTA resin. Gel filtration (Superdex‐200) was used for additional purification of recombinant HA. Fractions containing trimeric and higher order oligomers of HA with HA activity were pooled, concentrated, and stored at −80°C in 100‐mM Tris (pH 8)/150‐mM NaCl/10% glycerol.

### 4.5 Measurement of antibody binding by ELISA

Briefly, Immulon 2HB plates were coated with inactivated influenza virus at 1 μg/mL overnight at 4°C. Antibodies were prediluted in assay diluent (PBS containing 0.05% tween‐20 [PBST] and 10% fetal bovine serum [FBS]) to 0.8 mg/mL, followed by serial twofold dilutions in duplicate across plate. Plates were incubated with the test antibody for 2 h at 37°C. After rigorous plate washes in a microplate washer, a secondary anti‐mouse IgG antibody conjugated to HRP (SouthernBiotech, Birmingham, Alabama) was added to wells at 1:5000 dilution. Plates were incubated with the secondary antibody for 1 h and washed, and ABTS/H_2_O_2_ peroxidase substrate (SeraCare, Gaithersburg, Maryland) was added to assay wells. After 30 min at ambient temperature, reactions were stopped with 1% sodium dodecyl sulfate, and OD_405_ values were captured on the Versamax microplate reader using Softmax Pro 7 software (Molecular Devices, San Jose, California). The assay endpoint was defined as the reciprocal of the highest serum dilution at which the mean OD_405_ value averaged ≥0.05 and the IgG titer was calculated from the starting concentration of 0.8 mg/mL.

### 4.6 HI assay

The HI assay was performed in 96‐well plates (U‐bottom) by a standard method, essentially as described previously^15^ using 0.5% chicken red blood cells suspended in PBS (pH 7.2).

### 4.7 Plaque‐reduction neutralization test

MDCK cells were seeded at 9 × 10^5^ cells/well in tissue culture‐treated 12‐well plates. Influenza virus was titrated to approximately 250 pfu/mL and incubated with a range of mAb concentrations starting at 80 or 100 μg/mL and four‐ or fivefold dilutions of that, in duplicates, for 1 h at 37°C before infection of MDCK cell layers. After 1.5‐h adsorption at 37°C with the plates being rocked every 15 min, virus inoculum was removed, and the cells were overlaid with a 50:50 mixture of 2X EMEM plaque media (Quality Biological, Gaithersburg MD) and 2.4% Avicel RC‐581 (FMC BioPolymer, Philadelphia, PA)^16^ with TPCK‐trypsin (Quality Biological) at a final concentration of 2 μg/mL. At 96 h, the plaque media were removed, and the cells were fixed and stained with 0.3% crystal violet/20% methanol. Percent neutralization was calculated for each mAb concentration compared with no mAb controls.

### 4.8 Pseudovirus neutralization assay

Pseudovirus production and neutralization assays were performed as previously described.^17^ Briefly, full‐length HA open reading frames from H5 viruses (A/Vietnam/1203/2004, Indonesia/5/2005, A/Anhui/1/2005, A/gyrfalcon/Washington/41088‐6/2014, and A/duck/Bangladesh/19097/2013) and full‐length NA open reading frame from A/California/04/2009 were cloned into the pCMV/R expression vector. HA‐pseudoviruses carrying a luciferase reporter gene were produced in 293T cells by transfecting 5 μg of pCMVΔR8.2, 5.5 μg of pHR'CMVLuc, 0.5 μg of HA, and 4 μg of NA expression plasmids. HA‐pseudoviruses were collected 48 h post‐transfection, filtered through a 0.45‐μm low protein binding filter, and used immediately or stored at −80°C. HA‐pseudovirus titers were measured by infecting 293T cells with HA‐pseudoviruses for 48 h prior to measuring luciferase activity (luciferase assay reagent, Promega, Madison, WI) according to the manufacturer's instructions. HA‐pseudovirus titers were expressed as relative luminescence unit per milliliter (RLU/mL) of HA‐pseudovirus supernatants. Pseudovirus neutralization assays were performed using 293T cells in 96‐well plates. Pseudoviruses with titers of approximately 10^6^ RLU/mL of luciferase activity were incubated with serially diluted sera for 1 h at 37°C prior to inoculation onto the plates that were preseeded 1 day earlier with 3.0 × 10^4^ cells/well. Pseudovirus infectivity was determined 48 h post inoculation for luciferase activity. The inverse of the sera dilutions causing a 95% reduction of RLU compared with control was reported as the neutralization titer (ID_95_). Titers were calculated using a nonlinear regression curve fit (GraphPad Prism Software Inc., La Jolla, CA, USA). The mean titer from at least two independent experiments, each with intra‐assay duplicates, was reported as the final titer.

### 4.9 Competition ELISA

Competition ELISA was set up essentially as described previously.^11^ Briefly, ELISA plates were coated overnight with 2 μg/mL of soluble A/duck/Bangladesh/19097/2013 HA in PBS‐based coating buffer (SeraCare, Gaithersburg, Maryland). Unlabeled mAbs were added to the wells in duplicate, starting at a concentration of 500 μg/mL, and were serially diluted on the plates and incubated for 2 h at 37°C. Purified mAb 5C2 was labeled with HRP using EZ‐Link Activated Peroxidase Antibody Labeling Kit (ThermoFisher Scientific) and used as a detection antibody in competition with unlabeled mAbs at a concentration of 2 μg/mL for 1.5 h at 37°C. ABTS (SeraCare) was used as enzyme substrate.

### 4.10 Selection and characterization of escape viruses

The selection of H5 escape virus mutants was performed in MDCK cells, essentially as described previously.^13^ Approximately 10^4^ pfu of virus was incubated with H5 mAbs over a range of concentrations from 80 to 0.156 μg/mL before infection of MDCK cells. Virus obtained at the highest mAb concentration was passaged again in the presence of a fourfold higher mAb concentration and the process repeated for up to three more rounds of selection, after which the escape mutants were sequenced and tested for reduced inhibition of neutralization by the mAb compared with the parent virus.

### 4.11 Biolayer interferometry experiments

All biolayer interferometry experiments were performed on an Octet Red‐384 system using reagents and supplies from the manufacturer (Sartorius Corporation, Bohemia, New York). Biolayer interferometry experiments used Dip and Read Ni‐NTA coated biosensors in a 384‐well plate format (tilted‐bottom microplates) with a baseline buffer consisting of Kinetics Buffer with 0.1% Tween/0.1% bovine serum albumin (BSA). Ni‐NTA biosensors were coated with His‐tagged A/duck/Bangladesh/19097/2013 HA ectodomain at 3 μg/mL (determined empirically to generate a response signal of ~0.35). The mAbs 6D9, 5C2, and 4C2‐stem were loaded into adjacent wells at a concentration of 30 μg/mL for initial saturating binding to HA and also loaded into a second set of wells at a concentration of 30 μg/mL to be used as the competing Ab in the assay. Loading time for the His‐tagged antigen onto the Ni‐NTA biosensors was 500 s, with a threshold set at 0.35 nm; loading times for mAbs, either as the first (saturating mAb) or second (competing mAb) antibody, were 600 s. The binning experiment was designed so that each antibody was used for saturation and competition against the other antibodies. All steps were performed at 30°C and at a shake speed of 1000 rpm. Data Analysis HT 11.0 software (Sartorius) was used to calculate the shifts in wavelength after antibody addition.

## AUTHOR CONTRIBUTIONS

J.P.W. designed the study. C.S., F.S., M.O., C.A.M., A.V., W.W., A.W., V.N.A, and C.L.P prepared the reagents and performed the in vitro and in vivo experiments. F.S., C.A.M, C.D.W., and J.P.W analyzed and interpreted the data and prepared the figures. J.P.W. wrote the manuscript with input, review, and concurrence of all authors.

## CONFLICT OF INTEREST STATEMENT

The authors declare no competing financial or nonfinancial interests in relation to the work described.

## Supporting information


**Figure S1.** Monoclonal antibody detection of influenza HA in Western blot analysis. Viral proteins from inactivated influenza viruses (A) A/duck/Bangladesh/19097/2013 and (B) A/gyrfalcon/Washington/41088‐6/2013 were solved by SDS‐PAGE under reducing and non‐reducing conditions and probed in western blots with the indicated mAbs

**Figure S2.** Locations of amino acid mutations in two independently derived 5C2 escape viruses

**Figure S3.** Alignment of A/duck/Bangladesh/19097/203 HA with A/barn swallow/Hongkong/D10‐11613212/2010 HA

## Data Availability

The authors declare that all relevant data supporting the findings of this study are available within the paper and its supporting information files.
