# Supplementary figures and images for: Neutralizing and protective murine monoclonal antibodies to the hemagglutinin of influenza H5 clades 2.3.2.1 and 2.3.4.4
Source: Influenza Other Respir Viruses. 2023 May 25;17(5):e13152. doi: 10.1111/irv.13152 (PMC10209644; doi:10.1111/irv.13152)

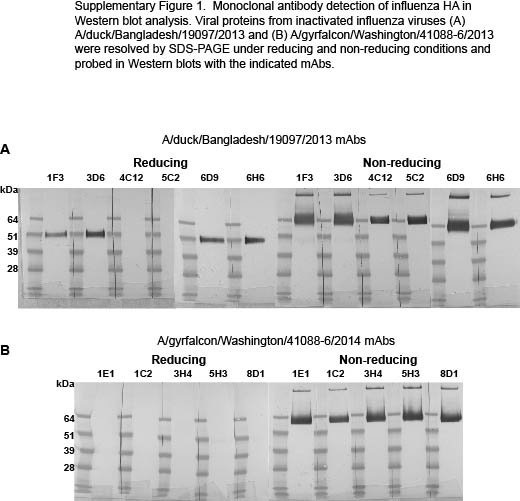

Supplement: Supplementary file 1 — Figure S1. Monoclonal antibody detection of influenza HA in Western blot analysis. Viral proteins from inactivated influenza viruses (A) A/duck/Bangladesh/19097/2013 and (B) A/gyrfalcon/Washington/41088‐6/2013 were solved by SDS‐PAGE under reducing and non‐reducing conditions and probed in western blots with the indicated mAbs [file IRV-17-e13152-s002.jpg]

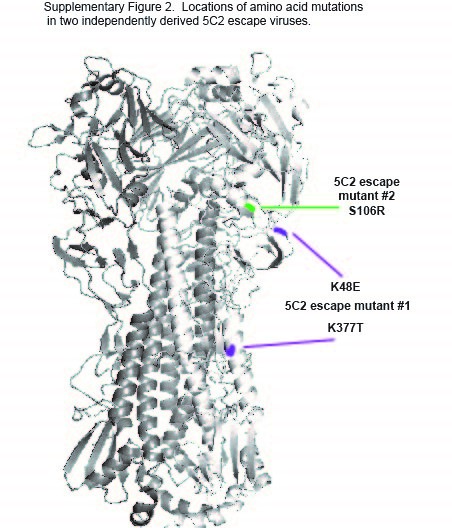

Supplement: Supplementary file 2 — Figure S2. Locations of amino acid mutations in two independently derived 5C2 escape viruses [file IRV-17-e13152-s001.jpg]

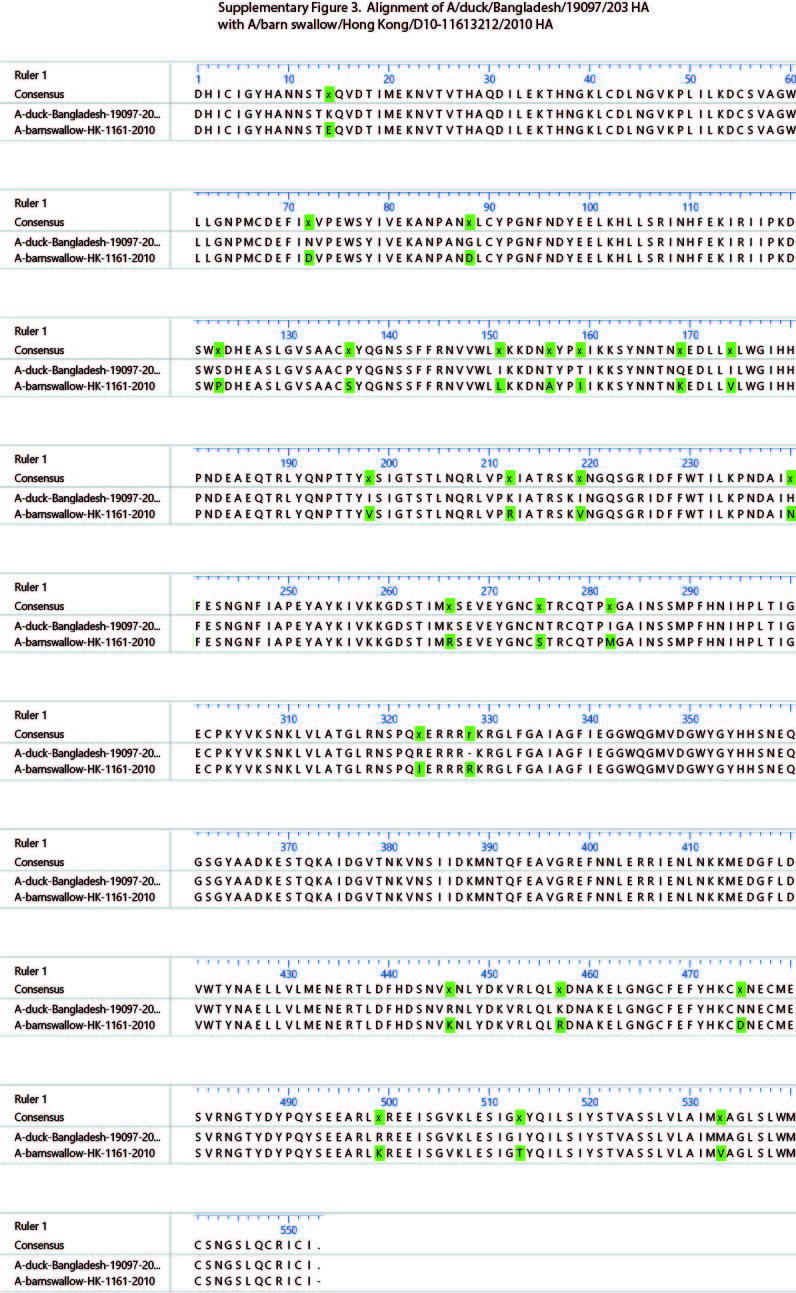

Supplement: Supplementary file 3 — Figure S3. Alignment of A/duck/Bangladesh/19097/203 HA with A/barn swallow/Hongkong/D10‐11613212/2010 HA [file IRV-17-e13152-s003.jpg]
